# What is known about persons with co-occurring problems’ experiences with supported housing, recovery, and health promotion? A scoping review

**DOI:** 10.1186/s12913-024-11736-z

**Published:** 2024-11-08

**Authors:** Unn Elisabeth Hammervold, Silje Gytri, Marianne Storm, Torgeir Gilje Lid, Hildegunn Sagvaag

**Affiliations:** 1 Department of Public Health, Faculty of Health Sciences, University of Stavanger, Stavanger, NO- 4036 Norway; 2 Faculty of Health Sciences and Social Care, Molde University College, Molde, Norway; 3 Research Department, Research Group of Nursing and Health Sciences, Stavanger University Hospital, Stavanger, Norway; 4 Center for Alcohol and Drug Research (KORFOR), Stavanger University Hospital, Stavanger, NO- 4068 Norway

**Keywords:** Co-occurring substance use and mental health problems, Supported housing, Recovery, Health promotion, Scoping review

## Abstract

**Background:**

Having a home is the foundation of most people’s lives. People with co-occurring substance use and mental health problems may experience challenges in acquiring and keeping housing. Many also have major health challenges. Supported housing is the subject of increasing interest, but there seems to be a lack of studies exploring supported housing’s potential for facilitating recovery and health promotion. Therefore, a scoping review was performed to answer our review question: *What is known in the literature about the experiences of persons with co-occurring substance use and mental health problems with supported housing*,* including experiences of recovery and health promotion?*

**Methods:**

Systematic searches were conducted in the Ovid MEDLINE, Embase, PsycInfo, CINAHL, Social Services Abstracts, Web of Science, Scopus, and Oria, and Idunn.no databases. The search terms were derived from the population, concepts, and context. The search for grey literature was conducted in various Norwegian sources.

**Results:**

Forty studies were included: 7 with quantitative design, 28 with qualitative design and 5 with mixed methods design. The studies were from Canada, Ireland, Norway, Scotland, and the USA.

The review identified four themes related to tenants’ experiences with supported housing:

1)The importance of a permanent and safe home; 2) Housing’s importance for physical health; 3) A shoulder to lean on – the importance of relationships and support; 4) the value of choice and independence.

Factors that may influence physical health were poorly represented.

**Conclusions:**

Long-term housing and safety are prerequisites for recovery for people with co-occurring problems. Programmes such as Housing First and Assertive Community Teams, especially, were experienced to support recovery. Autonomy was valued, including access to individual and respectful support from service providers when needed.

Supported housing may be a health-promoting arena, especially in relation to mental health. More attention should be given to how service providers can support tenants to protect their physical health, especially related to nutrition, meals and communal cooking. Further research is needed to tailor optimal services and support for people with co-occurring problems, including balancing support and autonomy with the aim of promoting health and recovery.

Peer specialists’ contributions to supported housing are scarce and need further development.

**Supplementary Information:**

The online version contains supplementary material available at 10.1186/s12913-024-11736-z.

## Background

Internationally, housing conditions are recognized as social determinants of health and well-being for people [1]. The right to adequate housing for all people was recognized as part of the right to an adequate standard of living in the 1948 Universal Declaration of Human Rights [2] and further stated in the Convention on the Rights of Persons with Disabilities (CRPD) in 2006 [3]. *Adequate* should include the right to live somewhere with security, peace, and dignity [4].

In 1986, the World Health Organization (WHO) defined health promotion as “the process of enabling people to increase control over, and to improve, their health” [5].

Income, housing, and food were among the factors acknowledged as primary prerequisites for health. The WHO also emphasized the need to provide information about life skills; create supportive environments that provide opportunities to make healthy choices; and create health-promoting conditions in economic, physical, social, and cultural arenas [6].

Homelessness, or inadequate housing, is a challenge in many countries [7, 8]. Persons with serious substance use problems and mental health problems (co-occurring problems) are especially vulnerable to the risk of being homeless and may experience difficulties related to housing on a recurring basis [8, 9]. In general, health, personal finances, social interaction, housing, personal relationships, and support are key influences on social recovery [10, 11]. Inadequate housing, poverty and a lack of support may thus seriously hinder recovery processes [12–14]. Anthony’s classic definition of personal recovery [15] has been criticized for its focus on recovery as an individualistic journey, neglecting the social, material, and cultural changes that influence peoples’ recovery processes [14]. Therefore, in this article, we lean on Topor et al.’s definition of recovery as “a deeply social, unique, and shared process in which our living conditions, material surroundings, social relations and sense of self evolve. It is about striving to live satisfying, hopeful, and reciprocal lives, even though we may still experience threats, stressful social situations, and distress. Recovery involves engaging in encounters and dialogues where new ways of understanding and handling one’s situation are created as we move beyond the psycho-social–material crisis” ([14], p. 11). In contrast to Anthony’s definition of personal recovery [15], this definition includes individuals’ social relationships and participation in community activities, thus acknowledging that personal and social recovery may intersect and influence each other. Consistent with this definition, we find Leamy et al.‘s conceptual framework for personal recovery relevant. It includes five recovery processes: connectedness, hope and optimism about the future, identity, meaning in life, and empowerment, which are collectively known by the acronym CHIME [16]. However, Stuart et al. [13] caution against an overly optimistic view of recovery from the service providers’ perspective. They argue that focusing solely on the positive aspects of CHIME may lead us to overlook the challenges individuals face in their recovery process. This narrow emphasis could lead care providers to homogenize experiences and blame service receivers for not making sufficient effort rather than creating an empowering environment. Therefore, they suggest extending the CHIME framework to include “difficulties”, resulting in the CHIME-D concept, which highlights the various challenges individuals may encounter during their recovery process. Examples of difficulties include financial concerns, negative life changes, and interpersonal problems [13].

People with co-occurring problems are seen by social and health services as individuals with a variety of challenges and needs [17]. Their life expectancy is 15–20 years shorter than that of the average population, a difference largely due to non-communicable diseases such as heart disease, stroke, cancer, diabetes, and chronic lung disease [18]. The picture is complex, however, and there may be several explanations, such as genetic vulnerability to mental health problems, factors related to health habits, psychosocial stress and loneliness, side effects of medications, illnesses, and harmful use of alcohol or other substances [18, 19].

The “deinstitutionalization” of mental health services, which started in many countries in the 1960s and 1970s, led to more people with mental health problems becoming homeless, especially in cases where planned growth in community support or other support did not happen [4]. Therefore, new policy approaches emerged across Europe and North America centred around service users and recovery, including providing housing and support services for persons with mental health and/or addiction problems.

A variety of housing approaches for persons with co-occurring problems can be found in the scientific literature. Examples include individual apartments or shared housing with various forms of support from the municipality [8, 20]. Trainor et al. [21] described the development of housing approaches as a continuum from *custodial housing* based on a medical model, usually in care homes with patients and care providers as stakeholders, to *supportive housing*, developed later and typically consisting of group homes or clustered apartments with residents and rehabilitation agents as stakeholders [21]. Unlike custodial housing, *supported housing* in the community is characterized by individual apartments, and the service users’ roles are tenants or citizens. In individualized follow-up services, housing staff serve as facilitators rather than rehabilitation agents [21]. Furthermore, in supported housing the staff focus on supporting the residents’ sense of choice and control, in line with recovery-oriented services [22, 23]. The Housing First (HF) model is described as particularly suitable for people with co-occuring problems. This model provides housing as quickly as possible to homeless people without any requirements for sobriety or being in treatment that have been a condition in traditional housing such as the treatment first model (TF) [24]. In addition to an apartment, the persons in HF programmes are offered treatment support and services such as *assertive community treatment* teams (ACTs) [20]. ACTs provide individualized community-based multidisciplinary services including medical, psychosocial, and rehabilitation services. The key elements of the ACT model are assertive engagement, delivery of services in the community, high intensity of services and holistic and integrated services [25]. ACTs and HFs are highlighted as models that have the potential to improve housing stability, increase service user satisfaction and reduce the use of health and social services [26, 27].

Recovery for people with co-occurring problems takes place in their everyday life, which implies an orientation towards supporting everyday solutions for everyday problems [23, 28].

Consequently, communities must facilitate services that support both personal and social recovery and promote citizenship [23]. Many people with co-occurring problems face challenges related to activities of daily living (ADL), such as cooking and cleaning. Supported housing may thus represent a supportive environment [5] where service providers can assist tenants with various health-related challenges by addressing the complexity of each person’s challenges related to co-occurring problems [29, 30]. In addition, the prevention and control of non-communicable diseases should be integrated into public services [18].

The aim of this study is to explore and summarize research findings, to map the status of knowledge and to identify research gaps related to the following research question: What is known in the literature about the experiences of persons with co-occurring problems with recovery and health-promoting factors in supported housing?

## Methods

A scoping review was used to identify, collect, synthesize, and summarize information to answer the research question. The scoping review follows the methodological framework developed by Arksey and O’Malley [31] and later improved by Peters et al. [32, 33]. We found the scoping review to be an appropriate method as it allows broad research questions and the inclusion of literature regardless of the methodological design. Following a scoping review framework, we carried out a five-stage process: Stage 1, identifying the research question; Stage 2, identifying relevant studies; Stage 3, study selection; Stage 4, charting the data; Stage 5, collating, summarizing, and reporting the results to comprehensively map the literature [31]. The process is described in the following sections.

### Stage 1: identifying review questions

In accordance with stage 1 of Arksey and O’Malley’s framework, we identified our research question. Owing to the nature of the research project, we initially aimed to focus solely on the supported housing experiences of individuals with both co-occurring problems and challenges related to violent behaviours. Our starting point was the master’s thesis research conducted by S.G. in 2020. A search of databases such as CINAHL, MEDLINE, SocINDEX, PsycInfo, Oria, and Idunn.no led to identification of 30 publications relevant to the aim of understanding the experiences and views of service users with co-occurring problems and violent behaviours regarding housing and support services.

The search strategy was developed further in collaboration with specialized librarians at the university and the Norwegian Institute of Public Health (NIPH). We decided to broaden our approach by omitting the term “violent behaviours” from the main search, as it did not yield relevant literature in scientific and professional databases. Smaller hand searches and consultations with professionals regarding violent behaviours also did not produce additional findings. Initially, we were interested in communal kitchens, but owing to the scarcity of literature on this topic, we expanded our focus to include health-promoting factors related to housing for individuals with co-occurring problems.

### Stage 2: identifying relevant studies

**Fig. 1:**
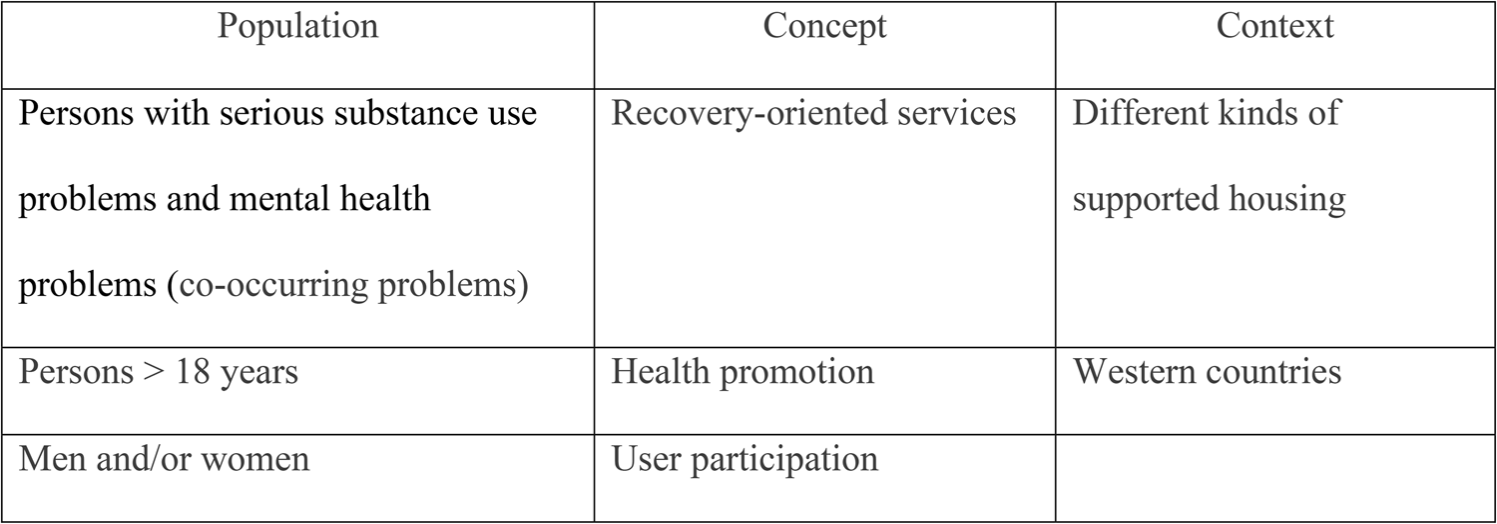
Overview of the Population-Concept-Context components

In stage 2, searches were carried out in Ovid MEDLINE, Embase, PsychInfo, CINAHL, Social Services Abstracts, Web of Science, Scopus, and Oria, and Idunn.no databases for peer-reviewed literature and grey literature. The search terms were selected on the basis of Population–Concept–Context (PCC) components [33], as shown in Fig. 1.

These components clearly define the scope of the review by specifying the studied population, the main concepts of interest, and the context in which the study is situated. The PCC components are recommended as a guide to develop a meaningful title and inclusion criteria, thereby providing a robust structure for the development of the scoping review [33].

The searches aimed to identify studies describing first-hand experiences with supported housing, including recovery and health-promoting factors.

The design of the search string was developed with support from a specialized librarian at NIPH and peer-reviewed by a second librarian before the authors carried out systematic searches in January 2022. The searches were repeated in October 2023. The searches in the academic databases focused on five main concepts combined with “AND”: (1) co-occurring substance-related and mental disorders, (2) housing with support services, (3) health promotion (including kitchen), (4) first-person experiences, and (5) research methods. The search terms under each main concept, including medical subject headings, terms, and synonyms for each main concept, were combined with “OR”. We did not set any limits for the year of publication, mainly due to our interest in mapping the existing literature on the topic. When searching for grey literature, hand searches were conducted in the Norwegian databases Oria.no and Idunn.no with different combinations of search words within the five main concepts.

### Stage 3: literature selection

In total, the searches in international databases yielded 6110 peer-reviewed articles once duplicates (*n* = 3040) had been removed. Additional publications from Norwegian databases were added to the peer-reviewed articles.

S.G. downloaded the search results into Rayyan.ai, an online screening tool, to organize them and facilitate collaboration in the screening process [34].

**Fig. 2:**
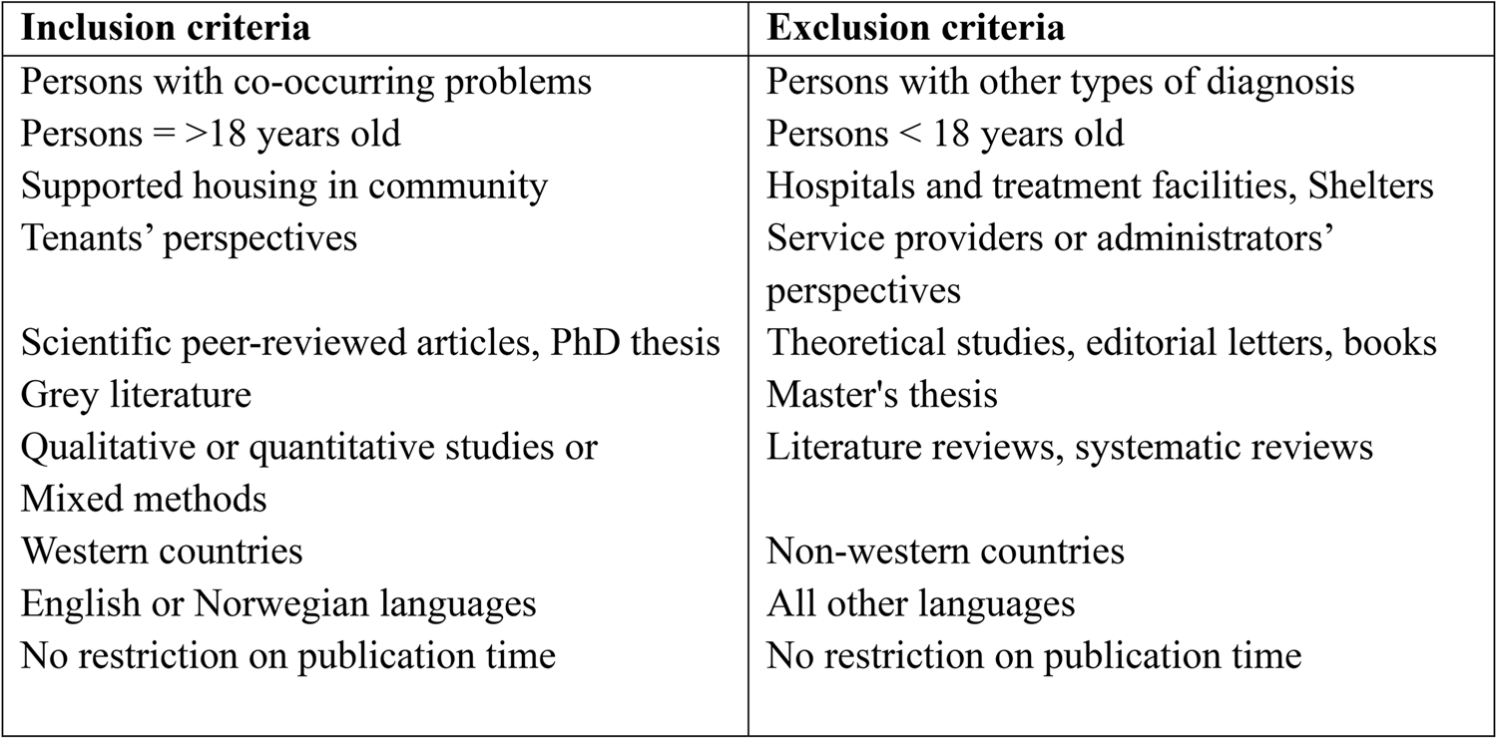
Overview of inclusion, - and exclusion criteria

Using the inclusion and exclusion criteria, shown in Fig. 2, the five researchers performed double-blind screening in Rayyan by (1) reading the publications’ titles, abstracts, and keywords and (2) reading the full texts. Publications were initially excluded on the basis of title and keywords. By reading abstracts, we were able to exclude publications on the basis of incorrect publication type; incorrect study design, e.g. reviews; an incorrect focus or context; inappropriate populations, such as children or youth; or participants without co-occurring problems. Full text publications were excluded based on inappropriate population, focus, context, or publication type.

Throughout the screening process, a third researcher facilitated consensus in instances where there was disagreement. Hand searches were conducted in the master’s thesis by S.G. and additional reference lists. Contacting 16 Norwegian professionals researching – or working in professional fields related to – co-occurring problems did not result in any new relevant articles [33].

**Fig. 3:**
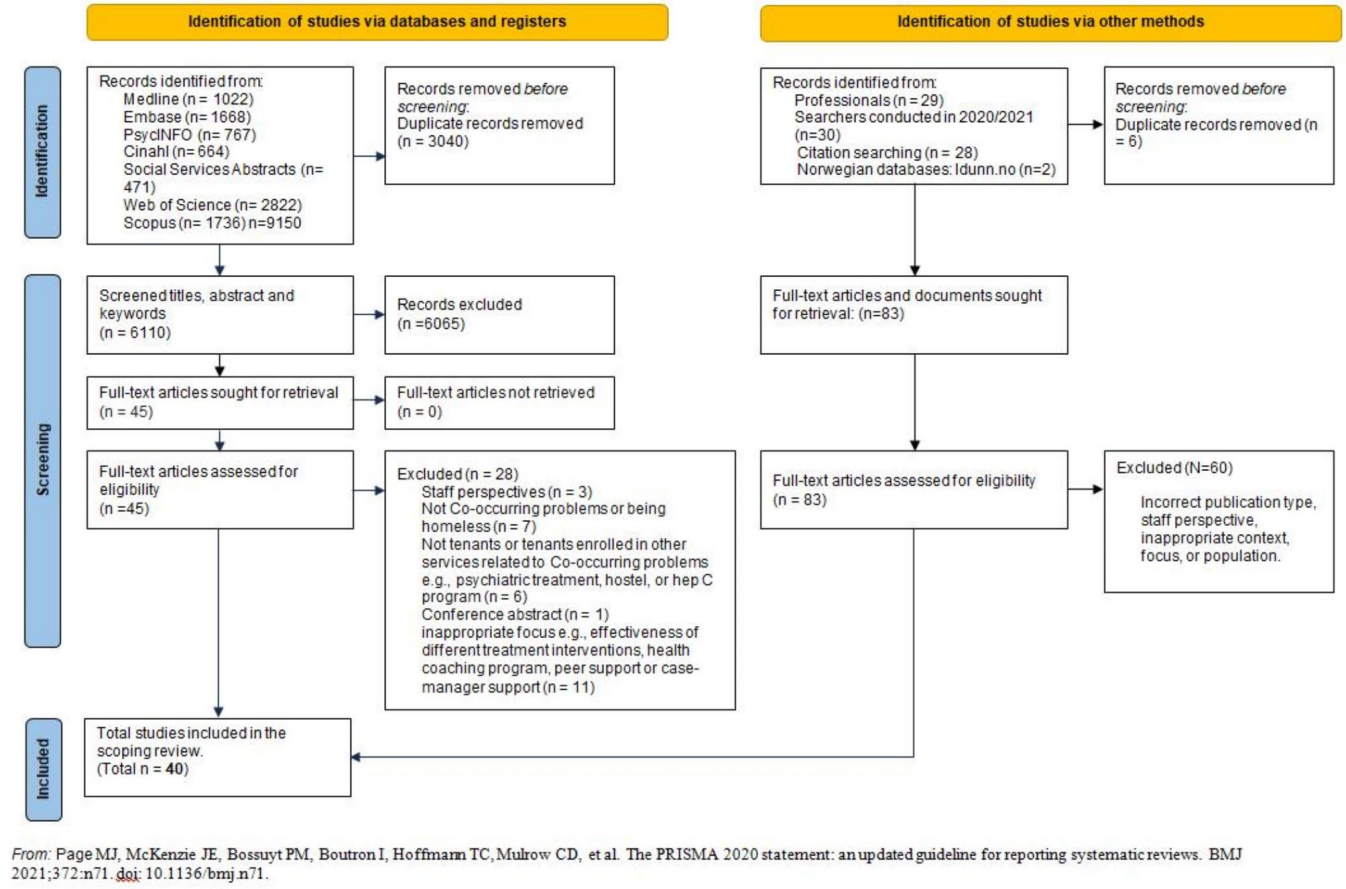
Study selection process

The overall screening process and selection are presented in the PRISMA flow diagram in Fig. 3.

The authors assessed a total of 40 publications for eligibility. Consistent with the scoping review methodology [33], we did not assess the quality of the included publications.

### Stage 4: capturing the data

Data related to the research question were extracted by S.G. and U.E.H. and reviewed by H.S. Key items from each publication were organized in a data charting form [31]. The form contained information about the first author, year of publication, study location, type of housing/programme, aims, methodology and findings. Table 1 provides an overview of the included publications.

**Table 1:** Overview of the included publications. *Grey literature

First Author	Study location	Type of housing and/or type of housing programme	Aims	Design/Methods	Findings
Andvig (2013)	Norway	Independent, scattered-sited permanent housing **Housing first program**	To highlight and describe experiences with making a home among persons with co-occurring problems	Qualitative design Individual and focus group interviews	Getting a safe home within supported housing and receiving support from staff and peers in daily life led to tenants feeling that they lived decent lives.
Andvig (2015)	Norway	Independent, scattered-sited permanent housing **Housing first program**	To explore, describe, and interpret service users` experiences of partaking in a housing first project (HF).	Qualitative design Individual interviews	When able to choose suitable supported housing on their own terms’, the tenants could start to recover and obtain a better quality of life. Being able to participate and to have choice in planning and formulating services was found to be important.
*Andvig (2016)	Norway	Independent, scattered-sited, and single-sited permanent housing. **Housing first program**	To evaluate HF project.	Qualitative design Individual interviews	Participating in the HF project improved living conditions and quality of life for a large proportion of the tenants. Getting a suitable place to live, having available and supporting staff as companions, and being able to take the lead in one’s own life were experienced as important in supported housing settings.
Bergman (2019)	USA	Independent, scattered-sited, and single-sited permanent housing. **Housing first program**	To investigate how emerging adults in two HF supportive housing programs describe and understand their experiences within these programs.	Qualitative design Individual and focus group interviews	Single-site housing: Positive experiences: having access to a variety of services in one place. Negative experiences: feelings of having staff and neighbors in “their business”, safety concerns related to drug use environment. Lack of quality and upkeep of the building. Scatter-site housing: Positive experiences: how quickly participants had been placed into their apartments. Negative experiences: safety concerns e.g., getting robbed. Overall, participants in both programs expressed appreciation for HF service providers.
*Bjørgen (2021)	Norway	Municipal rental apartments: single-sited temporary and permanent housing. **No housing program described**,** but recovery-oriented services**.	To carry out a survey to get feedback from persons with co-occurring problems about supported housing.	Qualitative design Individual interviews	Mixed experiences with supported housing including experiences with physical environment, social environment, substance environment and support services living in a single-site housing unit. Some study participants experienced feeling like they were living in an institution due to the way the housing unit were designed and due to surveillance. The findings suggest that there was a great potential for improvement for building relationships between tenants and providers, and for recovery perspective.
*Bordevich (2020)	Norway	Municipal rental apartments: single-sited temporary housing. **Individual Care Plan (IP)**	To gather experiences from persons with co-occurring problems about the preparation and use of Individual Care Plan (IP).	Qualitative design Individual interviews	How the Individual Care Plan (IP) was presented to the person mattered: good experiences with an easy-to-understand example in paper format. IP gave tenants a clear overview of their plans, goals, relevant contact information and who was responsible for different tasks. Positive experiences with having everything gathered in one document.
Carpender-Song (2012)	USA	Supported independent housing. **Recovery communities (RCs**): Structure and philosophy of supported independent housing.	To identify features of the contextual environment of recovery communities that contribute to recovery from the perspective of persons with co-occurring problems living in RCs.	Qualitative design Focus group interviews	Tenants living at the RCs experienced that the RC programs contributed to to their recovery by being part of a complementary service system, by providing a safe physical environment away from homelessness, drug activity and violence. RCs also provided a social environment with peer-support regarding mental health and sobriety.
Davidson (2014)	USA	Scattered-sited housing. **Housing First program (HF)**	To investigate the effects of HF implementation on housing and substance use outcomes.	Quantitative design Interviews at baseline and 12 months.	Tenants living in HF programs that were rated as user participation consistent, rather than user participation inconsistent, were less likely to be discharged. There was not found association between fidelity in implementation of supportive housing components and the tenants’ substance use. Persons in user participation consistent programs were less likely to report using stimulants or opiates at follow-up.
*Dyb (2015)	Norway	Municipal rental apartments: single-sited and scattered-sited housing. **Housing First program (HF)**	To determine the housing status of persons with co-occurring problems of low-threshold substance abuse programs.	Qualitative design Individual interviews	A variety of experiences with housing and support. Overall, tenants stated that having somewhere to live was important. There was a desire and a need for suitable housing and support at the right time for everyone. Feeling safe in the living environment seemed important to many tenants.
Friesinger (2019)	Norway	Supported housing: congregate apartments and co-located small houses. **No program described**	To contribute to improving the understanding of fire safety experienced by persons with co-occurring problems and how it is organized in supported housing.	Qualitative design multi-sited ethnography approach: field notes, interviews, and pictures.	Different experiences in supported housing connected to fire safety: positive (feeling protected) or negative (feeling annoyed or under surveillance).
Friesinger (2020)	Norway	Supported housing: congregate apartments and co-located small houses. **No program described.**	To explore how understandings of persons with co-occurring problems are expressed in the materialities of supported housing.	Qualitative design Multi-sited ethnography approach: field notes, interviews, and pictures.	Housing was seen as important. Location and feeling safe mattered. Living at a supported housing site made some tenants feel different than their neighbours. Descriptions, rules, and restrictions for using the common areas (including communal kitchen), and the fact that service providers had keys and power over these arenas: similarities between common areas and psychiatric wards. In their private space: it was important to be able to create a home, so it did not look like part of a hospital. Some had “vandal-safe” materials, this felt like “prison solutions”.
Gabrielian (2018)	USA	Different types of housing VA (Veterans) housing or **Housing First program** Men only	To examine the ways and identify the junctures in which service users` skills and deficits in accessing and mobilizing social supports (formal or informal) influence longitudinal housing status.	Qualitative design Individual interviews	Supports for maintaining housing: In stable, independent housing: formal (e.g., case managers) and informal (e.g., family) support, a predominance of informal supports that had emotional functions. Key informal support: family and twelve-step sponsors and peers. Sheltered housing: predominantly used formal support. A few tenants described informal support from peers for instrumental support (e.g., cleaning, cooking, mediating conflict with landlords/roommates/other tenants or managing finances) in housing setting. Unstable housing: predominantly used formal support. A few tenants described support from family for instrumental support in housing.
Greenwood (2017)	Ireland	Long-term homeless accommodation (more akin to “normal” living arrangements than to emergency or short-term accommodation) **No program not described**	To investigate consumer choice and mastery which is important to residential stability and psychiatric functioning for adults with histories of homelessness. To investigate whether these relationships hold, even in the context of problem-related substance misuse.	Quantitative design Questionnaires	Choice in housing and services were important to the tenants’ recovery experiences. Housing services should not take away user choice when a person’s substance use causes problems. Removing user choice can undermine a person’s psychiatric functioning.
Hansen (2020)	Norway	Supported housing Ambulant services, not specified	To explore if supported housing can be a solution for dual diagnosis clients?	Qualitative design Participatory observation and interviews	For tenants, permanent housing was a goal. Experiencing a sense of security and a fixed framework were positive. Communal areas were emphasized as an arena for fellowship where common activities and common meals were important. Service providers gave support to health and social services.
Henwood (2015)	USA	**Housing First program**: scattered-sited supported apartments or Treatment First (TF): temporary congregate shelter and services until a less structured placement e.g., single-room occupancy (SRO) or supported apartments	To examine differences in social networks of service users newly enrolled programs that use either a Housing First (HF) approach or a treatment first (TF) approach.	Mixed methods design In-depth qualitative interviews (baseline, 6 months, and 12 months). Quantitative and qualitative analysis of the data.	HF tenants had a greater portion of service providers in their social networks when compared with TF tenants. TF tenants were more likely to maintain mixed-quality relationships (relationships with both elements of support and conflict) than HF tenants. HF tenants viewed housing as providing a stable foundation from which to reconnect and restore social relationships. At the same time HF tenants were guarded about close relationships for fear of being exploited by others because they had gotten apartments. TF tenants were less inclined to develop new relationships with others (peers and service providers) because of temporary nature of living in a TF program.
Henwood (2018)	USA	**Housing First program**: scattered-sited supported apartments or Treatment First (TF): temporary congregate shelter and services until a less structured placement e.g., single-room occupancy (SRO) or supported apartments	To better understand contextual and environmental factors that influence health risk behaviors.	Qualitative design Ethnographic shadowing	A variety of experiences with contextual and environmental factors in tenants’ living environments. Both isolation and social engagement were chosen as approaches to reduce risk (e.g., using drugs, negative episodes with visitors at home) by tenants. Social engagement occurred in public rather than in the tenants’ private spaces. Some experiences with limited or lack of provider support and experiences of having to be their own caseworker. Experiences with the social milieu of their building and neighborhood and how the environment and identity can be tied together.
Knight (2014)	USA	SRO hotels **Women only**	To explore how SROs can operate as “mental health risk environments” in which macro-structural factors (housing policies shaping the built environment) interact with meso-level factors (social relations within SROs) and micro-level, behavioral coping strategies to impact women`s mental health.	Qualitative design Interviews Ethnography	Experiences that the physical environment matters and affect mental health: newly built SROs in contrast to older SROs were often clean, well-lit, less chaotic, well-managed, and safer. Sometimes, tenants had individual bathrooms and small kitchens to prepare food. Physical conditions that affected the tenants` mental health in the study included the presence of rats, mice and bed bugs, graffiti and broken furniture, and non-operating things (sinks, electricity, door locks and TV sets). Safety was mentioned as important in housing. Social isolation was used by some to reduce risk for victimization within unsafe living environments. The presence of hotel managers as support was highlighted as positive.
Lincoln (2009)	USA	Congregated housing unit. **Safe Haven shelter program (Housing First philosophy)**	To understand more about the lives and experiences of this group of chronically homeless adults and, more important, both what facilitated their coming into the Safe Haven and what their challenges were as they exited homelessness by coming into a Safe Haven.	Mixed methods design Qualitative individual in-depth interviews (3 and 9 months after entry) Quantitative data collection (at baseline, 6, and 12 months).	Descriptions of getting a place to live, and the importance of having privacy and one`s own key. Some aspects of the Model of Safe Haven were highlighted as positive: overall, the rules at Safe Haven were manageable meaning much less restrictive than other shelters. Service providers had respect for tenants` need for independence and treated tenants as adults. Safety was seen as important in housing. Trust and adjustment to living inside with others took time after years as homeless.
Manning (2019)	Ireland	Emergency or transitional housing **Continuum of care model: Congregate homeless services**	To test the hypothesis that choice in housing and services would predict recovery in several domains, and that these relationships would be mediated by mastery.	Quantitative design Survey data	Choice was important to recovery experiences among tenants` living withing congregate homeless services. Choice predicted mastery, which, in turn, was negatively related to psychiatric symptoms and drug use, and positively related to physical health and community integration.
Miler (2015)	Scotland	Unclear housing: Scattered sited HF program? **Housing First with intensive support or treatment as usual**	To address the gap in understanding the experiences and views of those providing and receiving HF tenancies and aimed to generate information to improve the service offered to clients and support offered to staff.	Qualitative design Individual interviews	Overall, positive experiences with housing and support within the HF program. HF had enabled positive changes in their life by making them feel safe, more motivated, independent, and wanting to get life “on track”. The support took place in everyday life. Descriptions of a breadth of supported offered: help with insurance, licenses, and applications; shopping and cooking; appointments; or support to reconnect with social network. Descriptions of challenges experienced by tenants: e.g., some expressed an unmet need for assistance with housekeeping, using appliances, cooking, time management and keeping appointments or budgeting.
Nelson (2015)	Canada	Housing not described. **Housing First programs or treatment as usual** (no housing or support provided through the study, but treatment as usual participants could use mental health and housing services available in their communities)	To compare the life changes of participants in Housing First and in treatment as usual from baseline to 18-month follow-up and examined factors related to various changes.	Mixed methods design Qualitative individual interviews. Qualitative and quantitative analyses. Interviews coded across 13 life domains (categorized: positive, mixed-neutral, or negative changes).	More than double the percentage of tenants in HF reported positive changes compared to tenants in treatment as usual, who were four times as likely to report negative changes. Good quality housing, increased control over substance use, positive relationships and social support and valued social roles were factors related to positive changes. Precarious housing, negative social contacts, isolation, heavy substance use, and hopelessness were factors related to negative changes.
Nesse (2020a)	Norway	Single-sited housing units or individual apartments. Supported housing programs.	To explore residents` self-reported recovery and quality of life and examine the relationships between these factors and issues in supported housing.	Quantitative design Questionnaires	Core issues in supported housing: staff support, housing satisfaction, sense of home, and satisfaction with personal economy were associated with both quality of life and recovery.
Nesse (2022b)	Norway	Single-sited housing units or individual apartments. **Supported housing programs.**	To examine the potential benefits of using a collaborative approach to recovery-oriented practice development for self-reported recovery and citizenship among residents at a supported housing site.	Quantitative design Prospective comparative design	Facilitating collaborative approaches to developing recovery-orientated practices can help promote recovery and protect citizenship for tenants in supported housing.
Ogundipe (2020)	Norway	Single-sited housing. Supported housing programs.	To explore how persons with co-occurring mental health and substance abuse problems, living in supportive housing, experience belonging?	Qualitative design Individual interviews	All themes in the study illustrated that the tenants’ community and contextual factors contributed to experiences of belonging. An individual’s experience of belonging is related to their surroundings e.g., unsafe/safe living environments and the degree of choice regarding where one lives.
Padgett (2012)	USA	Type av housing unclear **Housing First or Treatment First (TF) programs.**	To examine two strikingly different approaches to service provision for homeless adults with serious mental illness.	Qualitative design Individual interviews and observation recorded by interviewers.	The findings yielded four themes: (1) The impact of cumulative adversity on gender roles, social relationships, and a ‘normal’ life course. (2) Tenants’ experiences with services revealed individual acts of kindness within a system of care predicated on control. (3) Case managers had discordant priorities and relationships across the two approaches (HF vs. TF). 4) The benefits of permanent housing extended beyond residential stability. Recommendations for practice include respecting individuality, being sensitive to previous traumas, and working to achieve housing security sooner rather than later.
Patterson (2015)	Canada	Independent and congregate settings, homeless and precariously housed. **Housing First programs** or **treatment as usual.**	To examine key themes from narrative interviews conducted with homeless adults with mental disorders 18 months after random assignment to Housing First with intensive supports or to treatment as usual (no housing or supports through the study).	Qualitative design Individual interviews	Stable housing and participation in HF program led to positive changes across multiple domains for most of the participants in contrast to TF program participants. The sense of security associated with stable housing was the most influential factor that supported change.
Pettersen (2020)	Norway	Independent housing (*n* = 9) Supervised housing (*n* = 2) **Assertive community treatment (ACT)**	To explore experiences and perceptions of good quality housing from the perspectives of persons diagnosed with co-occurring problems and enrolled in a Norwegian program of assertive community treatment.	Qualitative design Individual interviews	Tenants described former living situations that were driven primarily by treatment providers and housing availability, as compared to their current living situations, which reflected a considerably higher degree of consultation and choice. The participants valued a secure housing situation, a balanced configuration of support and independence, and satisfying living environments. These factors were facilitated by enabling the participants to exercise choice over their housing situation. The study findings suggest that the development of successful housing programs for persons with co-occurring problems depends on their inclusion in housing planning and decision-making processes about where to live and when and how treatment providers should be present in their home.
Pringle (2017)	USA	Supported housing, not specified. **Housing First program merged with Integrated Dual Diagnosis Treatment (IDDT).**	To identify potential roadblocks to the functioning of a Housing First program when implemented in the context of IDDT and vice-versa.	Mixed methods design Qualitative individual interviews Quantitative measures	The permanency of supportive housing seems to be important to tenants to achieve educational and vocational advancement (IDDT stages).
Schutt (2021)	USA	Supported housing. Housing and Urban Development-**VA Supportive Housing program (HUD-VASH)** Weekly contact with peer specialist **Behaviour model**	To test hypotheses predicting use of substance abuse and mental health services and residential stability and evaluate peer specialists’ impact.	Quantitative design. Randomized trial of peer support added to standard case management in HUD-VASH. Measures: Average VA service episodes for substance use and mental illness; residential instability; preferences for alcohol, drug, and psychological services; extent of alcohol, drug, and psycho-logical problems; availability of a peer specialist.	Self-assessed health needs, mediated by service preferences, and assignment to a peer specialist predicted use of VA behavioural health services and residential stability, as did chronic medical problems, sex, and race. Conclusions: The behavioural model identifies major predictors of health service use and residential stability, but must recognize the mediating role of service preferences, the differing effects of alcohol and drug use, the unique influences of social background, and the importance of clinical judgment in needs assessment. Service availability and residential stability can be increased by proactive efforts involving peer specialists even in a health care system that provides services without a financial barrier.
* Skog Hansen (2017)	Norway	Individual apartments. **Housing First programs**	To evaluate Housing First projects in two municipalities	Qualitative design Individual interviews	The tenants reported a high degree of satisfaction with their housing. Individual follow-up and support were key factors enabling participants to live independently. The study was unable to identify any clear signs of improvement in the participants` mental health or substance use problems, or any concrete results in terms of the participants` recovery processes. But many of the participants strongly expressed their perception of improved quality of life after entering the program – having a secure place to live being crucial and there is a need for social inclusion to prevent loneliness.
Skog Hansen (2018)	Norway	Individual apartments. **Housing First programs**	To look at how Housing First service users perceive services and their relationships with service providers.	Qualitative design Individual interviews	Experiences with receiving social support with emphasis on user’s choice and self-determination as part of a Housing First program. The program practice was not entirely freedom of choice for the tenants, but a greater respect of their knowledge, perspectives, and opinions as a starting point for interventions. Tenants and service providers engaged in joint reflection work.
Tsai (2009)	USA	A variation of supervised housing, apartment housing, and SROs. **Different residential programs**	To determine (1) whether housing preferences differ by stage of treatment, (2) whether clients who prefer certain housing types have preferences for certain characteristics and what the differences are between housing types, and (3) what is important to clients and what leads them to their housing.	Mixed methods design Questionnaires Individual interviews.	No specific stagewise housing preferences were found in the surveys, but during interviews some tenants broadly related their housing preferences to their recovery. Preference for supervised housing was associated with structure and service provider and peer support while preference for apartment housing was associated with autonomy and privacy. Tenants in single room occupancies had the least choice and lowest satisfaction. Service provider recommendations and availability of housing were major factors in where participants were housed.
Tsai, (2010a)	USA	A variation of supervised housing, apartment housing, and SROs. **No program described**	To examine whether (a) housing preferences differ between stage of treatment for substance abuse, (b) tenants who prefer certain housing types have preferences for certain characteristics, and (c) tenants living in different types of housing report differences in social support, choice, and housing satisfaction.	Quantitative design Questionnaires	Most participants preferred their own apartment or house across different stages of treatment. Preference for supervised housing was associated with on-site care provider and peer support while preference for apartment housing was associated with autonomy and privacy. Tenants in single-room occupancies reported the least choice and the lowest satisfaction.
Tsai, (2010b)	USA	Supervised or independent housing arrangements. **No program described**	To examine housing preferences, decision making processes surrounding housing choices, and perceived barriers to housing.	Qualitative design Individual interviews	Tenants experienced that their housing preferences had changed over time, and some related housing preferences to recovery. Most tenants preferred independent housing, but many also described benefits of supervised housing. Tenants’ current living situations appeared to be driven primarily by service provider recommendations and availability of housing. Common barriers to obtaining desired housing were lack of income and information.
*Vassenden (2012)	Norway	Municipal housing: single-sited or scattered-sited housing. **No program described**	To explore how dwelling location—specifically, its relation to social housing dwellings and ordinary neighborhood dwellings—affects the integration of service users.	Qualitative design Individual interviews	Tenants had experiences with short term, unsafe and unstable housing environments in the past. A variety of experiences with housing and housing support services in relation to current housing experiences: both positive and safe, and negative and unsafe experiences.
Yuan (2023)	USA	Housing status variation (i.e., living in private or congregate housing settings, currently staying at homeless shelters, or precariously housed) **ACT services**	To elicit housing stability definitions from individuals with co-occurring problems and their behavioral health treatment providers How do individuals with co-occurring problems receiving ACT services and their ACT service providers define housing stability?	Qualitative design Individual interviews Focus group interviews	Development of a conceptual framework with two domains of subjective housing stability: functional stability and experiential stability. The functional stability domain includes four theoretical concepts: meeting basic needs, housing quality, housing affordability. and housing permanence. The experiential stability domain includes four theoretical concepts: autonomy and independence, connectedness, safety, and supportiveness.
Watson (2012a)	USA	Permanent supportive housing, both in one location and scattered site. **Housing First or Continuum of Care (CoC) programs.**	To develop a deeper understanding of the effect that the organization of mental health services offered in community setting has on the recovery process.	Qualitative design Focus groups and individual interviews	Experiences with COC programs: majority of the participants describe that tenants could either choose to follow a very defined and sometimes confusing list of rules and stay housed or break these rules and be evicted. The rules made tenants feel powerless and without control over their lives. Experiences with negative participant-care provider relationships due to the focus care providers placed on substance use and participation in CoC programs. Experiences with HF programs: Tenants met with care. Having choices was one of the most important parts of HF programs. Tenants did not get punished for substance use and felt secure in the knowledge that their housing was permanent. Experiences with positive participant-service provider relationships due to being able to speak openly about substance use. Due to experiences with CoC in the past, trust could take time for tenants even in a non-judging HF environment.
Watson (2012b)	USA	Permanent supportive housing, both in one location and scattered site **Housing First programs**	To address a gap in the research by studying the recovery process for formerly chronically homeless individuals with dually diagnosed serious and persistent mental illness (SPMI) and substance use disorder who are living in Housing First programming.	Qualitative design Focus groups and individual interviews	The recovery process in the programs was a negotiation between mental health and illness that tenants engaged to attain the highest quality of life possible despite symptoms related to their diagnosis. The structure of mental health services was key to this process, as it is often the policies that guide programming that determine access to the resources that are necessary for tenants to engage in this negotiation.
*Wågø (2019)	Norway	Municipal housing: single-sited or scattered-sited housing. Recovery oriented services Two of three housing communities: Men only	To test how housing quality and location affect residents with substance abuse and mental disorders and their living situation.	Qualitative design Focus group and individual interviews	There was not much difference between the housing preferences of tenants with co-occurring problems, and those found in the rest of the population. An additional finding is that there should not be a contradiction between a thoughtful and robust choice of materials and creating a homelike atmosphere. Furthermore, there should be a range of accommodation types (cohabitation and individual) that suit different requirements. Choosing who is allocated a place in a larger group is also important. A housing cooperative with several residents who have problems with narcotics, mental illnesses, and extroverted behavior requires 24-hour staffing to encourage residents and the environment around them to feel safe. Another important finding is that several residents expressed a wish to contribute to upgrading and repairing their homes.
*Wågø (2021)	Norway	Municipal housing: single-sited or scattered-sited small housing. **Recovery oriented services**	To collect and systematize the experiences with “small housing units” as housing for people with substance abuse and mental disorders (ROP disorders or combined disorders).	Qualitative design Questionnaire, interviews, and a film “Master in their own house?”.	Tenants considered it important to have a place that was theirs that was warm and where they could lock the door. The dwelling’s layout, location and expression were also important. This can support emotions such as hope, dignity, pride, and faith in the future. When location and appearance are perceived as sloppy and hidden, it can contribute to the opposite: the feeling of being given-up upon, stowed away or outside society.

### Stage 5: collating, summarizing, and reporting

In addition to organizing the quantitative and qualitative information into the data charting form, we analysed the qualitative publications using the data analysis software NVivo 12. To further analyse the data, we examined publications related to the research question to identify tenants’ experiences, applying Braun and Clarke’s thematic analysis [35].

This approach adopts a flexible theoretical stance and involves organizing the data into identified themes.

S.G. and U.H. collaborated on the analysis via NVivo. S.G. conducted the initial analysis by reading the articles to become familiar with the data, noting initial thoughts, ideas, and emerging codes. Based on the research question and PCC concepts, meaningful elements, such as quotes and descriptions of the emerging themes were identified, listed and grouped into potential themes in NVivo (e.g., experiences as homeless, views on different housing models, relationships, support, possibilities, difficulties). U.H. reviewed these potential themes in light of the research question and initial codes, and the two researchers discussed the further development of the themes. Preliminary themes were subsequently presented to the entire research group. The themes were discussed and redefined, and S.G. and U.H. created a complementary descriptive summary of the results.

## Results

### Study characteristics

Overall, 40 publications published from 2009 to 2023 were included, of which 30 were peer-reviewed articles, two were doctoral theses and eight were reports. Twenty-eight publications had a qualitative design, five had a mixed methods design, and seven had a quantitative design. Ten publications were written in Norwegian [36–45], and the rest were written in English. The study locations included Canada, Ireland, Norway, Scotland, and the USA. Three publications included participants from supported housing for men only [38, 43, 46] and one included participants from supported housing for women only [47]. Information about the participants’ sex was missing in five of the included studies [42, 45, 48–50].

Information about age was missing in nine of the included studies [24, 38, 42, 43, 45, 47, 49, 51, 52]. For the remaining studies, all the subjects were at least 18 years old. From the descriptions of the contexts, however, we concluded that the participants in all the studies were at least 18 years old.

The sample sizes of the included studies varied from *n* = 5 [51] to *n* = 358 [53]. In one of the included studies, information about sample size was missing [54].

Two studies were RCTs. Nelson et al. [55] compared the life changes of people participating in the HF model or the TF model. Schutt et al. [56] tested hypotheses predicting substance use, the use of services, and residential stability and evaluated peer specialists’ (individuals with lived experience) impact on these factors. In the studies with a quantitative design, outcomes were measured in different ways; consequently, it was not possible to pool the results into a meta-analysis. Therefore, quantitative findings are included in the narrative descriptions of the findings.

In the included literature, there were variations in the type of housing programme and type of housing: temporary housing, such as single-room occupancy (SRO) hotels and emergency shelters; transitional housing; permanent housing; housing with support staff; housing without support staff; HF and TF models; recovery communities; scattered-site housing; and single-site housing units. The type of housing model and support services used varied with respect to professional and value-related standpoints. Table 3 summarizes the 40 included publications, with the HF model being the most common, either as a single programme or combined with other programmes [24, 37, 40, 44, 49, 53, 55, 57–65].

In four publications, HF models were compared with TF models [24, 57, 59, 62].

Three publications dealt with recovery-oriented philosophy [42, 43, 54] and two publications dealt with ACT teams [66, 67].

Four main themes were identified through the analysis: (1) The importance of a permanent and safe home, (2) Housing’s importance for health, (3) A shoulder to lean on – the importance of relationships and support, and (4) The value of choice and independence.

### The importance of a permanent and safe home

In 24 of the 40 included publications, tenants expressed the importance of having a permanent home [24, 36, 37, 39–45, 49, 50, 52, 55, 57–59, 61–63, 65–69].

The importance of a safe living environment was highlighted in nearly half of the studies and was considered essential for tenants’ mental health [36, 37, 39–45, 47, 48, 50, 54, 57–59, 61, 65–67, 69].

The tenants’ descriptions of housing, housing support and living environments varied in the included literature. In 14 of the 40 included publications, tenants expressed positive opinions related to their physical living environment [36, 37, 40, 42–44, 48, 50, 54, 55, 61, 62, 66, 67]. Their descriptions of a positive, safe living environment included factors such as having their own key to lock the door to their apartment, location, ability to protect themselves against “addiction pals”, the presence of service providers and housing support [36, 39–45, 50, 54, 57, 58, 60, 61, 65, 66, 70].

Several tenants expressed a preference for having ordinary people as neighbours, especially families with children. An environment was considered “normal” in opposition to the “noise, violence, bugs and chaos” that characterize many shelters and rooming houses [62]. In one study [47], women in an SRO hotel for women only emphasized the importance of being safe in relation to previous experiences with trauma.

Other tenants expressed different preferences in terms of proximity or distance to an urban environment or nature [44, 55]. Joining permanent supported housing programmes made life easier, enabled positive life changes and offered hope for the future [24, 36, 41, 42, 44, 51, 62, 64].

Negative experiences related to the physical living environment were expressed in 14 of 39 publications [36, 40–42, 44, 47, 48, 51, 55, 62, 69–72].

These negative experiences were largely linked to illegal drug activity surrounding the living environment. Fellow tenants’ behaviours related to substance use or mental health, visitors from the drug milieu, and the drug environment in or surrounding the housing units were factors tenants described as contributing to feeling unsafe [36, 39–42, 44, 47, 51, 62, 69, 71].

As an example, Ogundipe et al. [51] reported that when fellow tenants and the living environment make tenants feel unsafe, supported housing does not feel like a home, making it difficult for tenants to feel a sense of belonging. Other residents’ behaviours, for example being in shared hallways and causing disturbances with loud noises or banging doors, created stress. Consequently, tenants found it hard to invite friends over to their unit like one would be able to do in an ordinary home. Other negative experiences with living environments include dirty and old buildings, bugs, poor repairs, cracks in walls, or living in dark basement apartments with small windows [44, 55, 69, 72].

In three studies exploring Norwegian tenants’ experiences, the housing environments were compared to those of psychiatric hospitals or prisons [36, 42, 48] because of the presence of aluminium toilets and sinks and fuse boxes to prevent fires while cooking. Furthermore, the entrance areas of the buildings reminded the tenants of a cage, with cameras and an intercom system which tenants and their guests had to use to communicate with staff [36, 42]. These areas were thus experienced as stigmatizing and degrading. The tenants reported that staff controlled communal areas such as social areas and laundry rooms by keeping the keys. These locked doors disrupted daily activities and made tenants feel that they constantly needed to ask for assistance [36]. We found that the kitchen was mentioned in descriptions of the physical environment in several studies [24, 36, 39, 40, 42, 43, 47, 50, 64, 66, 69, 71, 72]. There were descriptions of individual kitchens as being barely operational, but many tenants did not mind because “they were served meals anyway” [50]. Service providers in the same study expressed a tension in relation to serving food to the tenants as this could increase their dependency.

In some houses, access to the kitchen was restricted as the doors were locked; thus, tenants had to find a staff member to lock up [36, 50, 65]. The study also described the use of rules and restricted access to the communal kitchen (and other communal areas) because some tenants had “left messes” or because of tenants’ previous violent acts. One tenant stated that they felt as though they were being “treated like a child” [50].

SRO hotels were reported to be the least preferred housing option [71, 72]. The physical housing quality was reported to be the lowest and tenants reported significantly less choice and lower satisfaction than tenants did in other types of housing [47, 71, 72].

### Housing’s importance for physical health

In 22 of the 40 included publications, the tenants talked about health-related issues. Given that the population in this review consists of people with co-occurring substance use and mental health problems, it is a given that the tenants have varying degrees of poor physical and mental health. The significance for mental health of having a home was described in most publications. However, experiences related to mental health were expressed indirectly by talking about security, autonomy, independence, support, motivation, and hope. By charting the data, we found that physical health and illness were scarcely described in the publications. However, the tenants shared some experiences regarding physical health, medication support, substance use, kitchen access, and food.

Medication support was mentioned in six publications [24, 37, 57, 62, 72] and was described both as informal coercion and control [24, 36, 62] and as practical support [37, 57, 72]. The following example is from Watson (2012, p. 135): “If I look at recovery for my medical condition, it’s going from a place where I’m not taking meds and continually getting sicker, to a point where now I’m 99.9% adherent, meaning I’m taking my medications every day, on time, as prescribed” [57].

Several tenants talked about housing as a potential arena for change in substance use. Some pointed to the colocation of dwellings as challenging because tenants would buy, sell and share substances and alcohol with each other. However, some tenants participating in housing first programmes or ACT support reported a reduction in substance use [44, 53, 55, 57, 61, 62, 66]. They credited this reduction to the security of having permanent housing [57] or supportive social contacts [55, 61].

In one study [57], safe sex was an issue discussed in a harm reduction group, including by Rodney, who “recognized that he needed to start engaging in safer sex” (p. 161).

Meals were a topic in 10 studies [36, 37, 42, 43, 45, 50, 61, 65, 72, 73], whereas nutrition was briefly mentioned in only two studies [37, 42]. Communal meals were represented mainly in the Norwegian publications, but their frequency varied from daily to weekly [36, 37, 42, 43, 45, 50, 73]. Some tenants called for instructions and guidelines for cooking [50, 61, 72].

Another issue tenants highlighted related to physical health was support that involved being reminded of, encouraged to attend, and sometimes followed to health services such as doctors and dentists [36, 44, 45, 58, 61].

Finally, in some studies the tenants mentioned support with starting physical training [43, 55, 58]. This support was not clearly described, but in one study service users and service providers participated in a cycling race together [43].

#### A shoulder to lean on – the importance of relationships and support

Fifteen of the 40 included publications included descriptions of tenants’ experiences of homelessness or stories of having lived in temporary accommodations which were described in negative terms [24, 37, 40, 44, 45, 52, 54, 57, 58, 61, 62, 64–66, 69, 72].

Temporary, single-site shelters and hostels, in particular, were described negatively.

[24, 40, 41, 62, 65].

The tenants shared that experiences such as homelessness, being evicted, or living in poor quality housing environments had made it hard to trust that their current supported housing situation would be permanent and supportive. Trust was seen as something that took time to build, even after moving into safe and permanent supported housing [52, 57, 61, 65].

Tenants spoke positively about their relationships with service providers in 16 publications [24, 36, 37, 40, 44–46, 49, 52, 54, 57, 58, 61, 63–65, 72].

A common feature of these houses, except one [72], was that the housing was affiliated with the HF philosophy or was recovery oriented.

The tenants said that their relationships with service providers and how the service providers acted toward them mattered to their well-being. Service providers were described as loving, caring, respectful, considerate, committed, easy to talk to, honest, genuine, open, attentive, understanding, humorous, and flexible; they acknowledge the service users and push them in positive ways [36, 37, 40, 44, 52, 61, 65, 72].

According to Nesse et al. [68], support from service providers was positively associated with recovery and life satisfaction. One tenant in an HF project [58] said, “It is like having a shoulder to lean on. This is necessary in order to come further in life” (p. 176). Friesinger et al. [48] reported that tenants wanted to be taken seriously and not merely be reduced to a psychiatric diagnosis in their interactions with support services.

The tenants also emphasized the importance of having support services available when needed [37, 40, 44–46, 54, 58, 63]. When a tenant had a tough time, it was important for the service providers to respond quickly because this gave the tenant a greater sense of peace and security [37, 58].

The tenants expressed a need for varied and flexible support services in everyday life [36, 38, 40, 41, 43, 44, 46, 49, 51, 57, 58, 61–63, 67, 69]. Some examples mentioned were needing help with “everything they asked for”, including going to IKEA, making phone calls, contacting welfare services, opening bills, gaining control over their finances, going to the doctor, becoming involved in work or physical activities or cooking [37, 44, 46, 51, 58, 61, 63, 67].

Fellowship with other tenants was described positively in six publications [37, 51, 54, 65, 69, 72]. In other publications, tenants reported isolation and loneliness [36, 37, 40, 43, 46, 55, 60, 62, 70].

Peer specialist support was featured in six publications, either as a recommendation for the future [36, 42, 58] or in relation to peer specialists as employees [40, 44, 56]. Tenants in Skog Hansen’s report [40] emphasized the value of peer specialists in the follow-up service. Schutt et al. [56] compared peer support in addition to standard case management with standard case management alone in a supported housing programme for veterans. They reported that the presence of peer specialists predicted increased both the use of health services and residental stability.

### Value of choice and independence

The possibility for tenants to make active choices regarding housing and services was emphasized in several publications [40, 42, 51, 52, 57, 66, 69, 71, 72, 74, 75].

Manning and Greenwood [75] reported that choice is centrally important to the recovery process among homeless individuals. Their results indicate that choices could predict mastery, which in turn affects “self-appraised physical health, psychiatric symptoms, drug use, and physical and psychological aspects of community integration” (pp. 151–152).

The possibility of choosing between several apartments was valued in several studies.

[40, 42, 51, 66, 72]. However, some tenants expressed limited or no choice when it came to what apartment they received and consequently felt trapped [36, 51, 62, 63, 69, 71, 72].

For example, one woman described “choosing a house and a community as a choice between the lesser of two evils, highlighting that the choices were inadequate, which in turn made her feel like she did not truly have a choice at all” ([51], p.128). Choosing where to live could connect individuals both to other people and to the community. Some tenants expressed a need for a more independent life, which they could achieve by owning their apartment [51, 66]. However, they expressed the need for practical support from service providers regarding finances when it came to buying a residence or keeping their current apartment [37, 51, 66, 67].

Choices regarding the interior of their living space allowed them to add a personal touch, and they could also choose to contribute to the community, for example, by creating gardens around the buildings [37, 66]. In Andvig’s study [37], some tenants experienced being excluded from decisions related to the interior or colours of their living space. One tenant shared that refurnishing his apartment was a strategy that helped him avoid using illegal substances when the environment remined him of milieus he previously frequented. Other tenants emphasized how furnishing their home with personal things became a symbol of identity and personal history [66]. In contrast, tenants in another study commented that “receiving a fully furnished apartment made an enormous difference to their lives as they did not want to wait for deliveries of furniture, or to be put in an empty house. Some commented that receiving a fully furnished apartment was less stressful” ([61], p.5).

Autonomy and privacy were emphasized in four studies [24, 65, 66, 71]. The tenants in Lincoln et al.’s study [65] reported that they were required to stay overnight only two nights per week, and consequently they could choose to stay out or with friends overnight. Tenants in a housing programme with ACT support [66] emphasized their experience of privacy, ownership of their apartment, and gaining some kind of autonomy in their living situation. However, some tenants in a TF programme reported that their accommodations did not provide the privacy they desired. For example, an older tenant reported that service providers could come any time to take urine tests or that they gave him his money “when they [felt] like it” ([24], p.190).

Davidson et al. [53] reported that persons in programmes with greater fidelity to user participation and components of a housing first model had a better chance of staying in the housing programme. These service users were less likely to leave and reported using fewer opiates or stimulants at follow-up than those in programmes with inconsistent user participation.

When comparing TF housing with HF housing, Watson [52] found that the strict rules applied in the former model limited tenants’ agency; consequently, they did not “have control over their own lives” (p.335). In contrast, tenants in the HF housing model reported having choices, which was crucial for taking responsibility and creating meaningful lives.

## Discussion

When investigating the existing literature about people with co-occurring problems and their experiences with recovery and health-promoting factors in supported housing, we identified the values corresponding to different positions: *the importance of a permanent and safe home* is a material value, *housings’ importance for health* is both material and existential values and *choice and independence* and *a shoulder to lean on* are existential values.

### Physiological and security needs in everyday life

Because recovery occurs in everyday life [28, 76], it is essential that homes be permanent and safe, as highlighted in this review. The type of supported housing tenants are in significantly impacts living conditions, material surroundings and social relationships, which are crucial for creating a meaningful life [14]. Therefore, housing type plays a vital role in tenants’ mental health and in their recovery process. The positive experiences of tenants in supported housing support the right to adequate housing promulgated by the UN, which encompasses the security of tenure; the availability of services, materials and facilities; affordability; and a suitable location [4]. Because people with co-occurring problems often experience injuries, including physiological, physical and sexual injuries [77, 78], a secure home is crucial for their recovery.

Additionally, having a permanent and safe home corresponds to several elements of CHIME [16] such as hope for the future and the potential for developing one’s identity. This is particularly significant for individuals with co-occurring problems, whose identities are often shaped by experiences of marginalization and discrimination [79].

Living conditions and material surroundings are significant for recovery processes [14]. Receiving a permanent and safe home can strengthen a tenant’s potential to develop a more positive identity, including a social identity [16]. Choices regarding furnishing and personalizing new homes further influence identity development. However, tenants’ opinions regarding decorating their living space varied, suggesting that it is essential to offer them the opportunity to express their preferences before moving in.

The adverse housing experiences, that were identified particularly in temporary shelters and SRO hotels deemed unsafe and substandard, could correspond to the difficulties in the CHIME-D model that hinder the recovery process for these individuals [13].

Living conditions have been identified as one of the social determinants of mental health [5, 80]. According to our findings, if certain basic needs are met [80], *a permanent and safe home* represents a health-promoting and recovery-supporting factor for people with co-occurring problems.

*Housing’s significance for physical health* is another finding in the literature, which includes experiences related to medication support, substance use, kitchen access and food. However, related findings were scarce in the included literature, which may imply a lack of attention to, or prioritization of, tenants’ health conditions. Since physical symptoms in this target group are often misinterpreted as psychological issues or a lack of adherence to prescribed medication [18], this can contribute to poorer physical health and early death. Notably, the scant mention of medication support in only five studies may reflect a significant research gap or a disregard for the importance of proper medication management for tenants with co-occurring problems within supported housing environments.

In addition to housing, nutrition stands as a fundamental pillar for health promotion [81]. While our findings predominantly address meal frequency, it is essential to consider the overall quality and balance of the diet to fully support well-being. While the included articles did not discuss communal cooking as a means to enhance skills, mood, self-confidence, and self-esteem, such activities can play a crucial role in personal development even though the evidence is preliminary and limited [82]. In contrast, the focus on the technical specifications of kitchens and access regulations may inadvertently diminish tenants’ self-esteem. Such an emphasis could also impede the growth of tenants’ nutritional and culinary skills, potentially restricting their capacity to undertake health-promoting lifestyle modifications.

Motivation plays a role in recovery processes for people with co-occurring problems in their efforts to achieve personal goals. According to self-determination theory [83], humans are active organisms that are intrinsically motivated to perform a variety of tasks. The theory highlights three fundamental human needs: autonomy, competence and relatedness to others [83]. According to Anthony Mancini [84], self-determination theory can be seen as compatible with the concept of personal recovery as both perspectives emphasize the value of autonomy. In the interest of enabling and empowering tenants in supported housing to direct more attention to their physical health, we consider internal motivation [83], and thus self-initiated actions, to be essential. In addition, information about health habits and supportive environments is necessary for providing healthy choices [5].

Our review highlights a significant yet underutilized opportunity for supported housing to serve as a venue for health promotion, particularly through activities such as communal cooking. Such activities not only foster a sense of community but also empower residents with valuable life skills that contribute to their overall well-being. The kitchen is also an arena for interaction, which brings us to the next topic of discussion.

### Social and relational needs in everyday life

The significance of relationships, described in terms of having *a shoulder to lean on*,* the importance of relationships and support*, corresponds with social relations in recovery [14] and the connectedness element in the CHIME framework [16]. As trust takes time, relationships, participation in community, love and friendship are social needs that are difficult to meet in temporary shelters and SRO hotels, as highlighted in the literature. The deficiency of these factors seemed to influence the extent to which tenants were able to trust permanent and supported housing when offered. Supportive factors for tenants include relationships with service providers over time, the availability of support in times of crisis and flexibility between emotional and practical support in their everyday lives. As positive relationships with service providers have been described mainly in houses affiliated with HF or recovery-oriented philosophies, they may mirror recovery-oriented values such as autonomy, choice, responsibility, empowerment, personal meaning and understanding [23].

Experiences related to relationships with employed peer specialists were found in only three publications [40, 44, 56]. According to Slade [23], peer support specialists are a prerequisite for recovery-oriented practices. On the basis of their expert-by-experience knowledge, peer support specialists can introduce alternative perspectives and solutions to those delivered by professional service providers. They have been found to support people with mental health problems in improving their self-management of general medical condition [85]. Because most supported housing facilities in this review adopt recovery-oriented philosophies and considering the documented positive impact for service users of employees with lived experience [86, 87], we suggest the potential inclusion of peer support specialists as employees in supported housing. To this end, it is imperative to conduct additional research on the role and utility of peer support specialists in supported housing for service receivers with co-occurring problems.

### Esteem needs in everyday life

Our review identified choice and independence as key factors promoting recovery and health in supported housing. Recovery-oriented services are predicated on the principle that providers should actively support service users in achieving their personal goals, as advocated by Slade [23]. This approach requires service providers to adopt an ethical stance, treating tenants with dignity, respect and esteem, as noted by Atterbury [88]. However, our findings reveal variability in service provider roles, which aligns with previous research [89, 90], suggesting a dual function as both rehabilitation agent and facilitator. Facilitation involves bolstering tenants’ subjective well-being, encompassing the CHIME elements [16, 23].

The HF model prioritizes choice and independence, recognizing housing as a basic human right and advocating for service users’ active participation in housing decisions [20]. Autonomy and privacy are instrumental in fostering empowerment and enhancing control over one’s health, thereby facilitating recovery [16]. Importantly, autonomy may also entail emotional and practical support as needed by tenants. Service providers often encounter challenges when tenants in vulnerable situations avoid or decline contact and support. Neither motivation nor recovery occurs in a vacuum: the social environment and the availability of support are important [14, 84]. In supported housing there should therefore be a focus on building supportive and social environments in which each tenant is intrinsically motivated to develop and flourish. This contrasts with enforcing extrinsic motivation, whereby a person changes behaviours to achieve external outcomes, for example, for rewards or to avoid sanctions [84], as some tenants in TF housing communicate.

As human beings, we are all dependent on each other [91]. However, autonomy is a central value in the CRPD [3], which asserts that all individuals have an equal right to autonomy and independence. This right necessitates tailored, individual support to varying degrees in daily life. Shared decision-making, involving a collaborative process between tenants and service providers [92], can be a powerful tool for empowering tenants through an almost equal partnership. The diverse approaches used by service providers, as highlighted in the findings, emphasize the need to refine the definitions of recovery-oriented practices in supported housing.

In our discussion, we emphasized that material conditions are a prerequisite for meeting existential needs. Fundamental material values, such as a safe home, and existential values, such as human rights, appear to reinforce each other.

### The strengths and limitations of this scoping review

Our research question was broad; accordingly, a scoping review was appropriate to obtain an overview of the research field and practices and to identify knowledge gaps [31]. A strength of this review is the researchers’ multidisciplinary background, which means that they represent different professional perspectives. During the data collection period, two specialized librarians at the Norwegian Institute of Public Health were involved in optimizing and peer review search strategies, in addition to conducting the systematic searches. Together with searching for publications from a comprehensive group of databases, this has added rigour to the scoping process and is therefore a strength. All five researchers were involved in including/excluding literature by screening blindly in Rayyan; we consider this a strength [93].

The inclusion of grey literature suggests a more random selection. However, we found that the results in these publications were consistent with each other and with the scientific publications.

A scoping review does not require quality appraisal [31], and this can be seen as a limitation. The findings were restricted to studies published in Norwegian or English, which may have excluded other relevant international publications. Another limitation can be linked to the different sociocultural contexts or welfare systems described in the included literature; it is necessary to bear this in mind, even if our research question focused on people living with co-occurring problems. Additionally, the included studies varied in terms of study content, size, method and design. We wanted to “paint a picture” in our study by presenting the results together as a whole. This may have led to a loss of important details such as the statistical power of the quantitative studies due to the integration of their findings into a qualitative analysis, or of other aspects and findings within each included study and must be seen as a limitation. Despite these limitations, our review offers valuable insights into the experiences of people with co-occurring problems living in supported housing. We also discovered what appears to be a knowledge gap in the existing literature about people living with co-occurring problems and their experiences with different supported housing settings.

Consequently, the results may be useful for policy makers, practitioners and receivers of services [31].

## Conclusions

In the literature on people with co-occurring problems and their experiences with recovery and health-promoting factors in supported housing, material and existential values represent two sides of the same coin. This scoping review reveals that long-term housing and safety are prerequisites for recovery for people with co-occurring problems. Programmes such as Housing First and Assertive Community Teams, in particular, were experienced as positive based on their individual-oriented and flexible approaches, in which service providers had a key role. Autonomy was highly valued, including access to individual and respectful support from service providers when needed. Supported housing can serve as a health-promoting environment, especially for mental health. The findings from this study have several implications for further research. Notably, we found that studies exploring service users with violent behaviour are lacking, highlighting the need to prioritize this area of research.

Further research is also needed to tailor optimal service and support for people with co-occurring problems, ensuring a balance between assistance and autonomy to promote health and recovery.

More attention should be given to how service providers can support tenants in supported housing to protect their physical health, especially related to the potential of communal kitchens. Few studies on peer specialists were identified even though they generally are seen as valuable contributors to recovery generally. Therefore, we consider that further research is needed on peer specialists’ contributions to supported housing.

## Supplementary Information

Supplementary Material 1.

## Data Availability

All articles that were used as basis for data are available online.
