# Supplementary material for: What is known about persons with co-occurring problems’ experiences with supported housing, recovery, and health promotion? A scoping review
Source: BMC Health Serv Res. 2024 Nov 8;24:1366. doi: 10.1186/s12913-024-11736-z (PMC11549848; doi:10.1186/s12913-024-11736-z)
Supplement: Supplementary file 1 — Supplementary Material 1. [file 12913_2024_11736_MOESM1_ESM.docx]

# Declarations

**Acknowledgements**

We would like to thank the Norwegian Institute of Public Health for the cooperation in planning and conducting the literature searches.

## **Funding**

The study is a part of a project founded by Stavanger University, Norway.

## **Availability of data and materials**

All articles that were used as basis for data are available online.

## **Authors’ contributions**

HS advanced the initial idea for the article. SG, HS and UEH planned and carried out the literature searches. All authors participated in the screening and inclusion process. SG and UEH carried out the data extraction phase, did the data analysis and drafted most of the manuscript with significant contributions from HS, MS and TGL.

All authors contributed with significant input to drafts and revisions. All authors have read and approved the final manuscript.

## **Ethics approval and consent to participate**

Not applicable.

## **Consent for publication**

Not applicable

## **Abbreviations**

ACT: Assertive Community Team

CHIME: Five recovery processes that are significant for personal recovery:

Connectedness, Hope and optimism about the future, Identity, Meaning in

life and Empowerment.

CRPD: Convention on the Rights of Persons with Disabilities

HF: Housing First

NIPH: Norwegian Institute of Public Health

PCC: Population – Concept – Context

SRO: Single Room Occupancy (hotel)

TF: Treatment First

WHO: World Health Organization
